# Socio-demographic and economic inequity in the use of insecticide-treated bed nets during pregnancy: a survey-based case study of four sub-Saharan African countries with a high burden of malaria

**DOI:** 10.1186/s13690-023-01075-6

**Published:** 2023-04-21

**Authors:** Werissaw Haileselassie, Ruth Adam, Mizan Habtemichael, Randy E. David, Nabel Solomon, Salle Workineh, Jemal Haider, Ayele Belachew, Wakgari Deressa, Guiyun Yan, Nigussie Assefa Kassaw, Daniel M. Parker

**Affiliations:** 1 School of Public Health, College of Health Sciences, Addis Ababa University, Addis Ababa, Ethiopia; 2 Chief of Epidemiology and Population Health Sciences, Detroit Health Department, City of Detroit 100 Mack Ave, Detroit, MI 48201 USA; 3 School of Medicine, College of Health Sciences, Addis Ababa University, Addis Ababa, Ethiopia; 4 Program in Public Health, College of Health Sciences, University of California at Irvine, Irvine, CA 92697 USA

**Keywords:** Equity, ITN, Malaria, Sub-Saharan Africa

## Abstract

Despite global investments in malaria eradication and mitigation efforts, including the dissemination of ITNs to vulnerable communities, the goal of widespread malaria control among pregnant women has yet to be realized in many African countries. One of the explanations forwarded for this is related to the adoption and regular use of ITNs by pregnant women. Based on the available DHS and MIS data from four malaria high burden African countries– according to WHO malaria report 2020– inequality was measured by applying both relative and absolute summary measures for the four dimensions of inequality: economic status, education, place of residence and region. By considering the number of subgroups in each variable, simple and complex summary measures were used.ITN utilization by pregnant women showed an increasing trend over time in all the four countries. There was also significant inequality (variability) in the ITN utilization among population groups. DRC, Mozambique and Uganda showed noticeable inequality that favors the richest population, whereas in Nigeria the inequality was observed among both the rich and the poor during different survey yearsIn conclusion, in all the four countries, there were significant regional variations or differences in ITN use among pregnant mothers across all dimensions of inequality in the survey years. Tailored cost-effective interventions could be considered to improve ITN utilization among pregnant women.

## Introduction

Malaria is a mosquito transmitted disease caused by one of five plasmodium species. It is a major cause of mortality and morbidity in tropical and subtropical regions of the world, where it is endemic to 90 countries, placing216 million people at risk [1, 2]. According to the WHO world malaria report, in 2020, malaria was responsible for 241 million cases and 627,000 deaths [2]. The vast share of malaria cases and particularly deaths, occur in Africa (93% of worldwide deaths). Nigeria reports the largest share of cases globally (27%), followed by the Democratic Republic of the Congo (10%), Mozambique(5%), and Uganda (4%) [2].

Pregnant women are a special group at high risk of malaria infection, and severe complications, primarily due to impaired glucose metabolism and cell-mediated immunity (CMI). Prevalent complications include miscarriage, stillbirth, preterm birth, anemia, and intrauterine growth restriction [1]. A total of 25 million pregnancies are at risk of malaria related complications in sub-Saharan Africa each year [1, 3]. Determinants of severe disease include poor immune health, gravidity, trimester of pregnancy and presence of co-morbidities [3].

Two common malaria preventative measures for pregnant mothers are the use of intermittent preventative treatment using anti-malarial medications, and timely utilization of long-lasting, insecticide-treated nets (LLINs).Use of insecticidal bed nets such as LLIN is found to be an effective public health tool for control of malaria, especially among under-five children and pregnant women (especially in Africa) — the two most vulnerable groups [4]. A recent review by Cochrane [5], and multiple recent studies [6, 7] have clearly demonstrated a strong correlation between the use of ITNs and reduction in stillbirths, increase in birth weight, and a reduction in anemia and parasitemia levels among pregnant women. In addition to their use among pregnant women and children, ITNs generally reduce vector density [8].

Despite global investments in malaria eradication and mitigation efforts, including the dissemination of ITNs to vulnerable communities, the goal of widespread malaria control among pregnant women has yet to be realized in many African countries. Two explanations have been forwarded. The first is sub-optimal distribution of ITNs. Only an estimated 17% of ITNs have been distributed in sub-Saharan Africa, the region of the world that is most greatly affected [3]. This has been blamed by the WHO on supply chain and logistics shortcomings [4]. The second explanation, though not well understood, is related to the adoption and regular use of ITNs by pregnant women. Relationships with health workers, cost, distance to distribution points, knowledge of prenatal care and local environmental and sociocultural factors have been proposed to influence ITN use [1]. Other explanations such as discomfort with ITN use, and perceived low mosquito density have also been proposed by researchers [9].

Addressing these challenges requires an informed multidisciplinary approach that includes (1) the acceleration of programs that integrate malaria prevention with maternal and reproductive health, (2) increased provisioning of resources to the most needy groups, and (3) the development of innovative delivery approaches (4).

As part of routine antenatal care (ANC), ITNs are used to prevent malaria in pregnancy. This tool has been proven to be highly effective in all parts of the world as an effective method of reducing human–vector contact and thereby decreasing morbidity and mortality due to malaria. Despite the presence of such effective malaria preventive tool, the prevalence of malaria among pregnant women in sub-Saharan countries remains high, resulting in significant morbidity and mortality. This needs characterization of ITN utilization among pregnant women for targeted intervention. Thus, the current study was designed to analyze ITN utilization in African countries with the high burden of malaria such as Nigeria, Uganda, Democratic Republic of Congo (DRC), and Mozambique. In light of this ongoing problem, the specific purpose of this study was to investigate sub-national and sociodemographic disparities in ITN use among pregnant women by using data from the Demographic and Health Surveys of 2003, 2008, 2011, 2013, 2016 and 2018, and Multiple Indicator Cluster Surveys. The findings of this study are believed to support national and regional efforts to accomplish the Sustainable Development Goals (SDGs) in the respective countries, which includes goals of universal health coverage and ultimately reduced inequities.

## Methods

### Setting

This study was conducted based on the Demographic and Health Surveys (DHSs) and Multiple Indicator Cluster Surveys (MICSs) from Nigeria, the Democratic Republic of Congo (DRC), Mozambique, and Uganda.

Nigeria is located in the tropical zone of West Africa, on the Atlantic coast, and has a total surface area of 923,768 km^2^. Nigeria is the most populous country in Africa with estimated populace of 225.1 million (2022) [10]. It is characterized by three distinct climate zones, a tropical monsoon climate, a tropical savannah climate, and a Sahelian hot/semi-arid climate. Mean annual temperature for Nigeria is 26.9 °C, with average monthly temperatures ranging between 24 °C (December, January) to 30 °C (April) [11]. According to a recent USAID report, 76% of Nigeria’s population reside in areas of high malaria transmission [12].

Even though transmission season is different from place to place, there is malaria transmission throughout Nigeria, where 97% of the population are at risk. There is year-round transmission in the south to three months or less in the north. As reported by World Malaria Report, in 2020, Nigeria had the highest number of global malaria cases and the highest number of deaths [13]. To reduce the malaria burden, Nigeria is implementing the following key interventions: insecticide-treated nets, targeted indoor residual spraying, intermittent preventive treatment in pregnancy, and effective case management [14].

The DRC is located primarily in central Africa and covers 2,344,858 km^2^, making it the second largest country in Africa, by area. Its climatic condition is tropical in equatorial river basin; cooler and drier in southern highlands; cooler and wetter in eastern highlands. The country’s estimated population is 108.4 million (2022) [10]. The DRC has a largely tropical equatorial climate; however, this varies across the country’s extensive area. Generally, the country is hot and humid in the north and west, an area located within a significant portion of the Congo River Basin. The southern, central and eastern areas are generally cooler and drier. Mean annual temperature for the DRC is 24.1 °C, with average monthly temperatures ranging from 24.6 °C (March) to 22.9 °C (July) [15].

In DRC nearly 97% of the population lives in zones with stable malaria transmission lasting 8–12 months per year with the highest transmission in the north and center. DRC government launched the High Burden High Impact initiative in 2019 to align interventions with disease burden in ten most affected provinces [16].

Mozambique is located in southeastern Africa and encompasses 799,380 km^2^of surface area. It has an estimated population of 31.7 million (2022) [10]. The climate is generally tropical, with a hot, rainy season between November and March, and a dry season between May and October [17].

Mozambique is among the four countries with the highest malaria cases and deaths worldwide (4.2% of global cases and 3.8% of global deaths in 2020). The country has the second highest prevalence of malaria in Eastern and Southern Africa (17.9% in 2020). According to MIS data from 2018, malaria prevalence is higher in the Northern and Central regions and lower in the Southern region [18]. Indoor residual spraying and Insecticide-Treated Net distributions (ITNs) are among the interventions implemented in Mozambique with the help of PMI program [19].

**Fig. 1:**
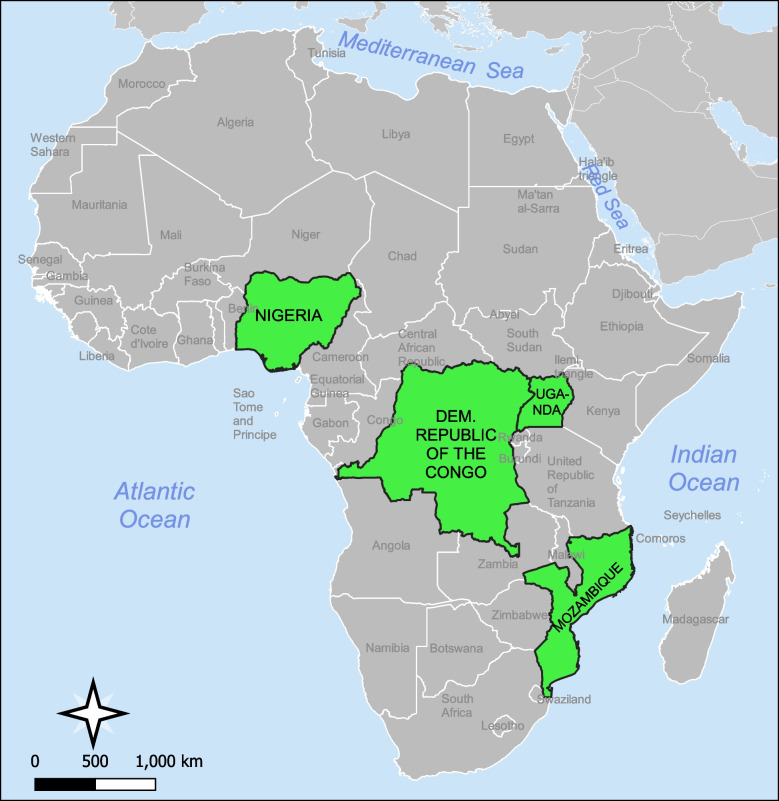
Map of Africa, with the countries of interest of this study in green

Uganda is located in East Africa and shares its western border with the DRC.It has a total surface area of 241,038 km^2^. Uganda’s estimated population is 46.2 million (2022). Its climate is tropical and generally rainy, with, however, two short dry seasons (December to February, and June to August); The far northeast of the country is semiarid [10]. Average temperatures range between 20 °C and 25 °C, with warmer temperatures occurring between December and March, and a relatively cooler period between June and September [17] (Fig. 1).

Uganda has a decreasing malaria transmission from 42% in 2009 to 9% in 2018; yet, in 2020, Uganda had the 3rd highest global burden of malaria cases and deaths (5.4%) and the 5th highest level of deaths (3.5%). There is stable, perennial malaria transmission in 95% of the country. By adapting mixes of interventions and other strategies, the Ugandan government, through Uganda Malaria Reduction and Elimination Strategic Plan 2021–2025, aims to reduce malaria infections by 50%, morbidity by 50% and mortality by 75% by the year 2025 [20].

### Data source

The World Health Organization’s Health Equity Assessment Toolkit (HEAT) was the source of all national DHSs and MICSs utilized for this study [21]. The surveys analyzed from each of the four study countries were conducted in different years. For Nigeria, data from 2003, 2008, 2011, 2013, 2016 and 2018 was used. For the DRC, data from 2007, 2010, 2013 and 2017 was utilized. For Mozambique, data from 2011 and 2015 was used. Lastly, for Uganda, data from 2006, 2011, and 2016 was utilized. Even though the survey seasons might have an impact on ITN utilization, all the DHSs data analyzed in this study have different survey periods between countries and between each survey year. Therefore, it is hard to assess the effect of seasonal variations on ITN utilization.

### Variables and measurements

For all of the study countries, the proportion of pregnant women sleeping under an ITN the night preceding a given survey was presented using four dimensions: economic status, education, place of residence, and subnational region. These dimensions were selected due to availability of disaggregated data on HEAT, and relevance with ITN utilization. Economic status was used to classify household living conditions. Data on a household’s ownership of selected assets, such as televisions and bicycles, materials used for housing construction, and types of water access and sanitation facilities were all included. Analysis of economic status, i.e. wealth index, was done using principal components analysis (PCA), and relative wealth was classified into five wealth quintiles [22]. Educational status was classified by three categories: (1) no formal education, (2) primary school (3) secondary school. Place of residence was categorized as urban or rural depending on each country’s national classification. Subnational region was classified according to the central government’s administrative system.

### Statistical analysis

HEAT version 3.1 software was used for statistical analyses. Six summary measures were calculated for each survey from the four countries of interest. Summary measures used to assess inequality were a combination of both absolute and relative measures: Difference (*D*), Absolute Concentration Index (ACI), Population Attributable Risk (PAR), Ratio (*R*), Relative Concentration Index (RCI) and Population Attributable Factor (PAF). The absolute measures (D, ACI, and PAR) indicate the magnitude of difference in health between subgroups and they retain the same unit as the health indicator, whereas the relative measures, R, RCI and PAF, show proportional differences in health among subgroups and have no unit [23].

### Description of summary measures

For education, economic status, place of residency and subnational region, *D* was calculated as the percentage of pregnant women sleeping under an ITN in the advantaged subgroup (highest wealth quintile, secondary education or higher, urban dwellers, or the subnational region with the highest estimate of pregnant women sleeping under ITNs) minus the percentage in the disadvantaged subgroup (no formal education, the poorest (lowest wealth quintile), rural dwellers,or the subnational region with the lowest estimate of pregnant women sleeping under ITNs). The *R* value was calculated in a similar way as *D*, except that division was used as the mathematical operation instead of subtraction.

PAR was calculated as the difference between the estimate of pregnant women sleeping under ITNs for the advantaged subgroups (as aforementioned) and the national average for the proportion of pregnant women sleeping under ITNs. PAF was calculated by dividing the PAR by the national average (*μ*) and multiplying the fraction by 100, i.e. [PAF = (PAR/μ) × 100].

To calculate *ACI*, the following formula was employed: *ACI* = ∑*jp*(2*Xj* − 1)*yj*, where (1) *yj* indicates the estimate of ITN utilization for each subgroup j, (2) *p* indicates the population share of subgroup j, (3)*Xj* indicates the relative rank of subgroup j, and relative rank is calculated as: (*Xj* = ∑*jpj* − 0.5*pj*), obtained from a weighted sample of the whole population rank from 0 (most disadvantaged subgroup) to 1 (most advantaged subgroup). RCI was calculated by dividing ACI by μ [24].

## Results

### ITN utilization by education attainment

**Fig. 2:**
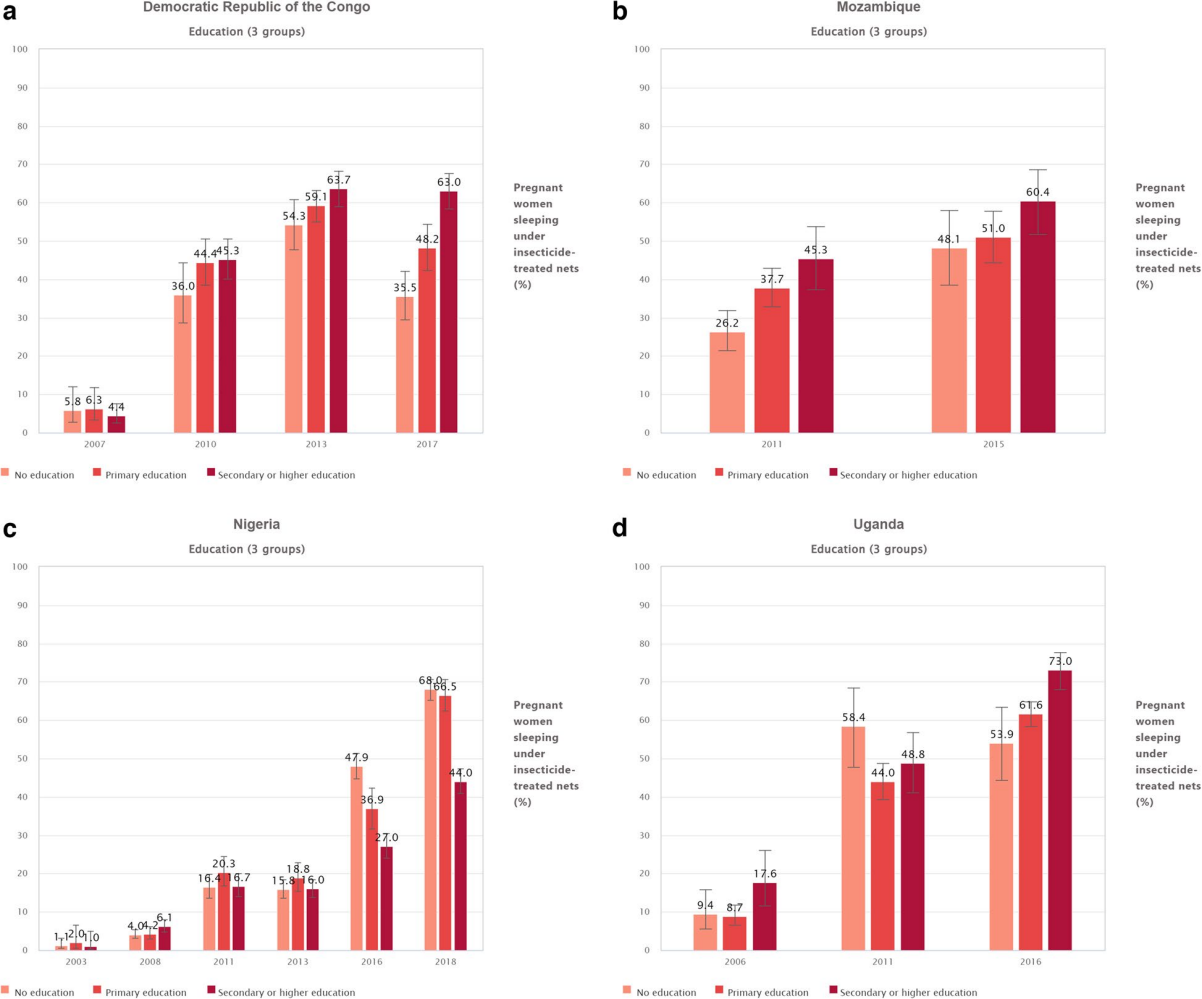
**a** Proportion of pregnant Congolese (DRC) women sleeping under ITNs, by educational attainment (DHS 2007, and 2013 and MICS 2010,and 2017). **b** Proportion of pregnant women sleeping under ITNs by educational attainment in Mozambique (DHS 2011, and 2015). **c** Proportion of pregnant women sleeping under ITNs by educational attainment in Nigeria (DHS 2003, 20,008, 2013, 2018, and MICS 2011, 2016). **d** Proportion of pregnant women sleeping under ITNs by educational attainment in Uganda (DHS 2006, 2011, and 2016)

As depicted on Fig. 2a, in the DRC, the proportion of pregnant women who slept under an ITN showed a sharp increase between 2007 and 2013. For instance, among the subgroup with no formal education, in 2007, the proportion of ITN use was 5.8% (95% CI: 2.7, 12); however, by 2013 it was 54.3 (95% CI: 47.6, 60.8). This upward trend occurred in all educational subgroups.

The proportion of pregnant women who slept under an ITN also increased in Mozambique across all education subgroups (Fig. 2b). As showed in Fig. 2c, in Nigeria, there was a growth in usage of ITNs in the survey years 2003, 2008 and 2011, but a slight decrease in 2013, followed by a sharp increase in 2016 among all education subgroups. In Uganda the proportion of pregnant women who slept under ITNs increased in the 2006, 2011, and 2016 surveys except for a potential slight decrease among those with no formal education (53.9 (95% CI: 44.2,63.3) in 2016 from 58.4 (95% CI: 47.7,68.3) in 2011) (Fig. 2d)*.*

### ITN utilization by urban/rural setting

**Fig. 3:**
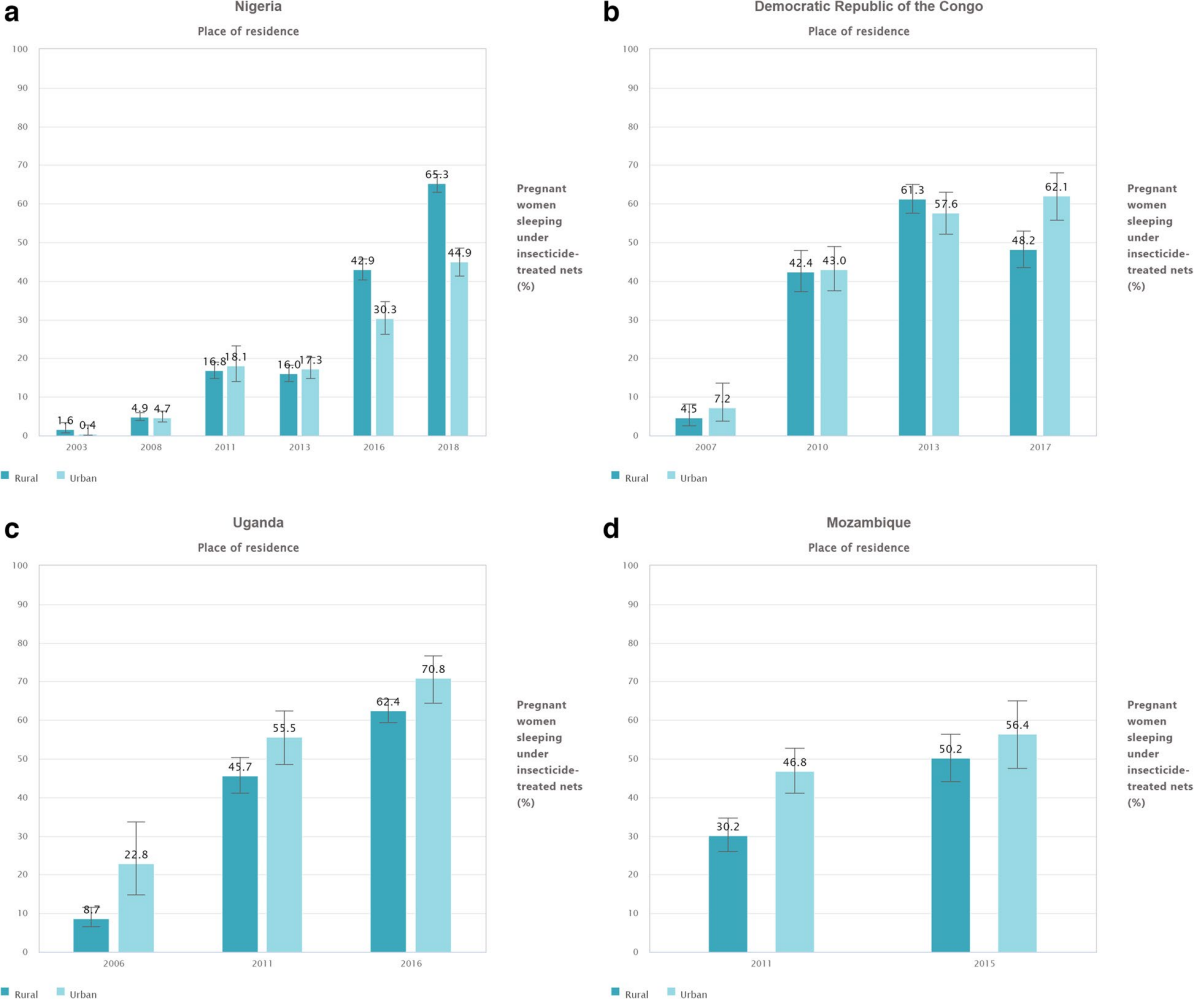
**a** Proportion of pregnant women sleeping under ITNs by urban/rural setting in Nigeria (DHS 2003, 20,008, 2013, 2018, and MICS 2011, 2016). **b** Proportion of pregnant Congolese (DRC) women sleeping under ITNs, by urban/rural setting (DHS 2007, and 2013 and MICS 2010,and 2017). **c** Proportion of pregnant women sleeping under ITNs byurban/ruralsetting in Uganda (DHS 2006, 2011, and 2016). **d** Proportion of pregnant women sleeping under ITNs by urban/rural setting in Mozambique (DHS 2011, and 2015)

In Nigeria, despite differing proportions of ITN usage across urban and rural settings, both settings exhibited an increasing trend in all years that surveys were analyzed except for 2013 (2003,2008,2011, 2016, and 2018). For example, in 2011, in rural regions 16.8% (95% CI: 14.8, 19.0) of pregnant women slept under an ITN. In 2013, it became 16.0% (95% CI: 14.0, 18.2). This trend was similar in the urban setting too (Fig. 3a).

In the DRC, the proportion of pregnant women who slept under an ITN increased in all analyzed surveys, except for the year 2017, when the rural population estimate decreased from 61.3% (95% CI: 57.5, 65.0) in 2013, to 48.2% (95% CI: 43.5,52.9), representing a noteworthy decrease (Fig. 3b).

In Uganda, ITN usage increased in both urban and rural settings in survey years 2006, 2011, and 2016 (Fig. 3c). In Mozambique, the trend was similar, with improvements in ITN utilization during survey years 2011 and 2015 (Fig. 3d).

### ITN utilization by economic status

**Fig. 4:**
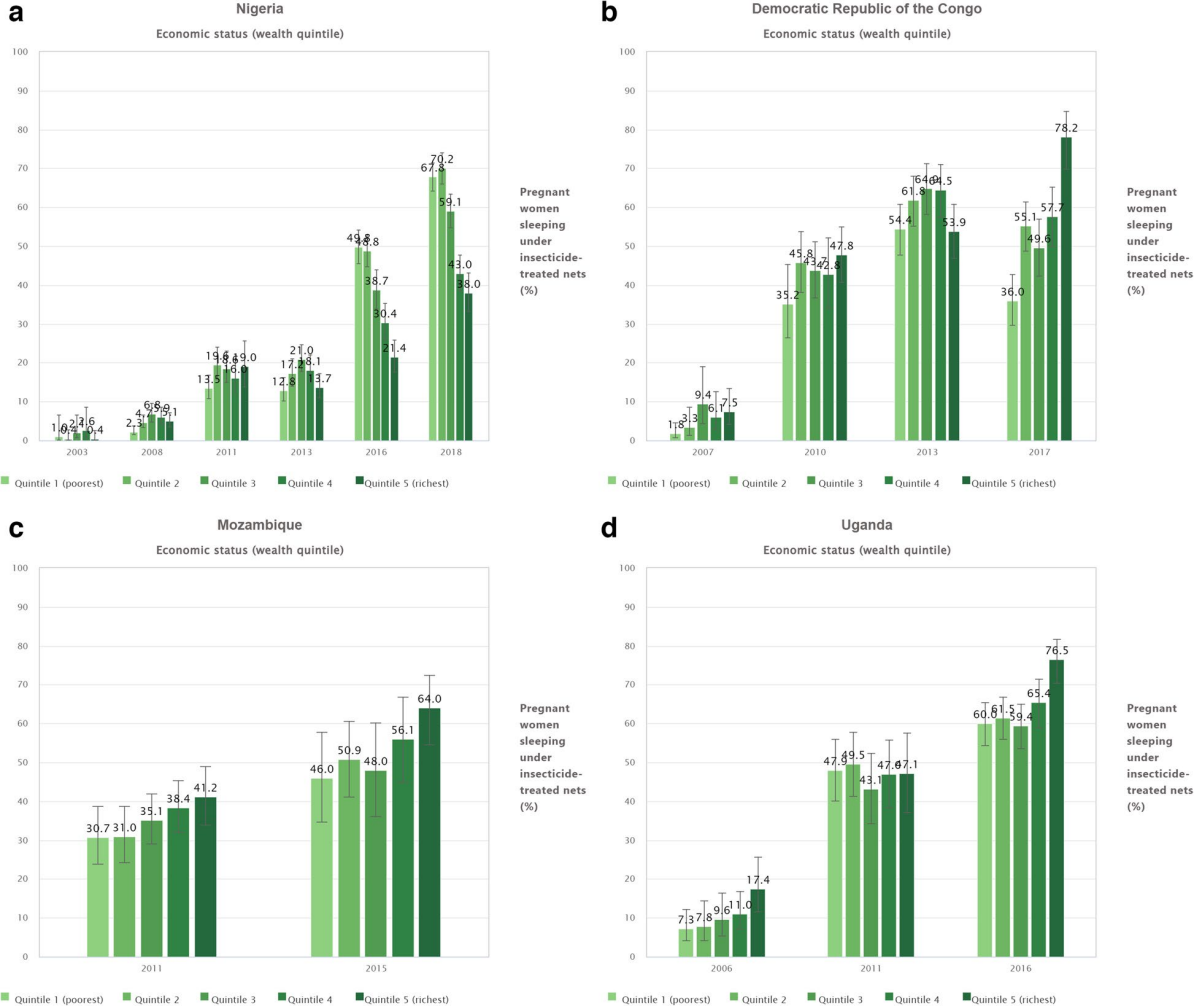
**a** Proportion of pregnant women sleeping under ITNs by economic status in Nigeria (DHS 2003, 20,008, 2013, 2018, and MICS 2011, 2016). **b** Proportion of pregnant Congolese (DRC) women sleeping under ITNs, by economic status (DHS 2007, and 2013 and MICS 2010,and 2017). **c** Proportion of pregnant women sleeping under ITNs economic status in Mozambique (DHS 2011, and 2015). **d** Proportion of pregnant women sleeping under ITNs by economic status in Uganda (DHS 2006, 2011, and 2016)

In Nigeria, during the years 2003, 2008, 2013,and 2018 all economic quintiles reported an increasing trend in the utilization of ITNs among pregnant women. For example, in the poorest quintile, ITN usage was 1.0% (95% CI: 0.1, 6.6) in 2003, and increased to 2.3% (95% CI: 1.4, 3.7) in 2008. In the following study years, 2013 and 2018, ITN utilization increased to 12.8% (95% CI: 10.1, 16.2) and 67.8% (95% CI: 64.1, 71.3) (Fig. 4a).

In the DRC, ITN utilization increased over survey years 2007, 2010, and 2013. In survey year 2017 it exhibited a decreasing pattern in all economic strata except for the wealthiest quintile. For instance, in the third quintile, ITN utilization was 9.4% (95% CI: 4.4, 19.0), 43.7% (95% CI: 36.6, 51.0) and 64.9% (95% CI: 58.1, 71.2) in the years 2007, 2010 and 2013 respectively,then became 49.6% (95% CI: 42.3, 57.0) in the year 2017 (Fig. 4b).

In Mozambique and Uganda, ITN utilization among pregnant women increased by passing survey years, across all economic quintiles (Fig. 4c and d).

### ITN utilization by subnational regions

For Nigeria (Tables 1 and 2), the DRC (Table 3), Mozambique (Table 4), and Uganda (Table 5), there was an increase in ITN usage across all subnational regions in all survey years.

**Table 1:** Proportion of pregnant women sleeping under ITNs in Nigeria, by subnational regions (DHS 2003, 2008, 2013)

Country	Survey Year	Subnational Region	Proportion of ITN Usage (%)	95% CI (Lower Bound)	95% CI (Upper Bound)
Nigeria	2018	north central	48.9	44.2	53.7
		north east	57.7	53.4	62
		north west	78.9	75.6	81.9
		south east	38.5	33	44.2
		south south	29.2	23.4	35.8
		south west	31.3	26.1	36.9
	2013	north central	15.8	12.3	20
		north east	13.2	10.2	16.9
		north west	16	13.3	19.2
		south east	23.4	18.3	29.4
		south south	16.6	12.6	21.4
		south west	18.7	14.1	24.3
	2008	north central	3.4	2.2	5.3
		north east	5.7	3.8	8.3
		north west	4.2	3	5.8
		south east	6.4	3.9	10.1
		south south	7.1	4.8	10.5
		south west	3.4	1.9	6.2
	2003	north central	1.6	0.2	10.7
		north east	1.7	0.5	5.6
		north west	1.1	0.3	3.6
		south east	1.5	0.2	10.1
		south south	1.5	0.2	9.2
		south west	0	0	0

**Table 2:** Proportion of pregnant women sleeping under ITNs in Nigeria, by subnational regions (MICS 2011 and 2016)

	2011			2016		
**Subnational Region**	**Proportion of ITN Usage (%)**	**95% CI (Lower Bound)**	**95% CI (Upper Bound)**	**Proportion of ITN Usage (%)**	**95% CI (Lower Bound)**	**95% CI (Upper Bound)**
Abia	10.1	4	23.4	11.3	5.3	22.6
Adamawa	21.1	13.8	30.8	48.5	37.8	59.2
akwa ibom	26.6	18.6	36.4	15.9	8.1	28.8
Anambra	17.5	9.7	29.5	10	3	28.6
Bauchi	15.9	6.5	34	60.4	53	67.3
Bayelsa	5.6	2.2	13.2	11.8	6.3	21.1
Benue	3.2	0.7	13	57.8	45.9	68.9
Borno	2.6	0.8	8	38.1	24.2	54.2
cross river	29.1	19.1	41.5	40.9	24.8	59.2
Delta	4.6	1.7	11.5	16	6.8	33.1
Ebonyi	7	3	15.3	29.1	18.5	42.6
Edo	11.2	3.9	28.2	4	1	15.4
Ekiti	40	26.4	55.3	12.4	3.5	35.7
Enugu	3.9	0.9	15.1	5.8	2	16
fct abuja	20	7	45.4	8.2	3.9	16.5
Gombe	48.6	32.4	65	54.9	43.1	66.1
Imo	1.8	0.3	11.7	9.4	3.6	22.3
Jigawa	28.6	19.5	39.9	63.4	50.7	74.4
Kaduna	37.1	26.8	48.7	22.5	15.9	30.9
Kano	26.3	19.4	34.6	51.1	44.4	57.8
Katsina	28.4	20.4	38	48.5	38.5	58.6
Kebbi	7.9	4.6	13.1	37.8	28.8	47.6
Kogi	12.1	4.9	27	9.4	2.4	30.7
Kwara	10	4	23.2	9.6	2	35.7
Lagos	4.6	1.5	12.9	4.1	1.7	9.7
Nasarawa	21.8	13.9	32.7	35	26.6	44.5
Niger	23.2	15.8	32.8	29.3	20.3	40.4
Ogun	12.8	6.7	23	10.3	3.7	25.5
Ondo	3	0.4	19.6	20.3	8.5	41
Osun	1.8	0.4	7.2	10.5	3.5	27.7
Oyo	15.1	7.3	28.6	15.3	7.8	28
Plateau	48.1	35.9	60.6	41.6	29.3	55.1
Rivers	29.7	14.6	51.1	11.6	4.3	27.5
Sokoto	6	2.8	12.2	40.4	30.7	50.9
Taraba	2.9	1	8	14.9	8.6	24.4
Yobe	14.5	3.9	41.7	66.6	55	76.4
Zamfara	2.5	0.9	6.8	66.1	58.4	73

**Table 3:** Proportion of pregnant Nigerian women sleeping under ITNs by subnational region in DRC (DHS 2007, 2013, and MICS 2010, and 2017)

Country	Survey Year	Subnational Region	Proportion of ITN Usage (%)	95% CI (Lower Bound)	95% CI (Upper Bound)
DRC	2017	bas uele	57.8	38.8	74.7
		equateur	52.3	41.8	62.6
		haut katanga	65.4	43.8	82.1
		haut lomami	71	56.9	82
		haut uele	65	43.7	81.6
		ituri	21.1	13	32.2
		kasai	22	14.4	32.1
		kasai central	45.1	35.3	55.3
		kasai oriental	35.1	20.3	53.3
		kinshasa	76	66.1	83.7
		kongo central	63	46.9	76.6
		kwango	48.8	33.1	64.8
		kwilu	71.9	60.5	81
		lomami	48.3	38.6	58
		lualaba	47.2	36.2	58.4
		maindombe	72	48.5	87.6
		maniema	51.6	37.1	65.9
		mongala	71.9	46	88.5
		nord kivu	31.4	21.4	43.6
		nord ubangi	65.1	56	73.1
		sankuru	23.8	10.8	44.7
		sud kivu	41.5	26.6	58.1
		sud ubangi	83.7	71	91.5
		tanganyika	37	23.8	52.5
		tshopo	71.9	58.6	82.3
		tshuapa	33.9	21.8	48.6
	2013	bandundu	79	73.6	83.5
		bas congo	65.5	50.4	78.1
		equateur	70.7	62.2	77.9
		kasai occidental	44.8	33.3	56.9
		kasai oriental	55	47.3	62.6
		katanga	65.3	58.6	71.5
		kinshasa	38.4	28.3	49.6
		maniema	54.8	42.4	66.6
		nord kivu	46.1	32.9	59.9
		oriental	44.2	33.6	55.3
		sud kivu	61.9	52.7	70.3
	2010	bandundu	42.8	32.6	53.7
		bas congo	32.9	22.9	44.7
		equateur	52.6	38.1	66.8
		kasai occidental	24.4	16.8	34.1
		kasai oriental	14.6	8.8	23.1
		katanga	35.2	24.3	48
		kinshasa	42.2	31.7	53.4
		maniema	66.6	56	75.8
		nord kivu	39.1	30.9	47.9
		province orientale	61.9	51.8	71.2
		sud kivu	56.6	43.9	68.5
	2007	bandundu	4	0.7	20.7
		bas congo	24.9	14.7	39
		equateur	5.2	1	23.4
		kasai occidental	2.1	0.4	9.7
		kasai oriental	9.3	3	25.8
		katanga	3.7	1.2	11.2
		kinshasa	6.9	3.2	14.4
		maniema	8.2	3.8	16.7
		nord kivu	6.8	1.1	33.2
		oriental	0.3	0	2.6
		sud kivu	8.1	2.8	21.2

**Table 4:** Proportion of pregnant women sleeping under ITNs subnational regions in Mozambique (DHS 2011, and 2015)

Country	Survey Year	Subnational Region	Proportion of ITN Usage (%)	95% CI (Lower Bound)	95% CI (Upper Bound)
Mozambique	2015	cabo delgado	66	48.8	79.8
		gaza	29.6	18.6	43.5
		inhambane	62.6	48.6	74.8
		manica	49.6	32.2	67.1
		maputo cidade	46.4	28.7	65.1
		maputo provincia	49.9	38.1	61.6
		nampula	53.2	41.5	64.6
		niassa	48.3	32.7	64.2
		sofala	70.6	59.9	79.3
		tete	53.3	40.6	65.5
		zambezia	42.1	25.7	60.5
	2011	cabo delgado	38.4	28.1	49.9
		gaza	8.7	4.5	16.1
		inhambane	33.5	23.4	45.4
		manica	39.2	30.8	48.2
		maputo cidade	33.7	25.3	43.2
		maputo provincia	23.9	16.9	32.8
		nampula	52.2	42.5	61.7
		niassa	36.2	27.8	45.4
		sofala	40.8	30.1	52.5
		tete	22.2	14.7	32
		zambezia	27.5	19.6	37.1

**Table 5:** Proportion of pregnant Ugandan women sleeping under ITNs by subnational regions (DHS 2006, 2011, and 2016)

Country	Survey Year	Subnational Region	Proportion of ITN Usage (%)	95% CI (Lower Bound)	95% CI (Upper Bound)
Uganda	2016	acholi	68.2	60.3	75.1
		ankole	60.6	50.4	69.9
		bugisu	68.8	57.8	77.9
		bukedi	46.1	36.8	55.6
		bunyoro	63.6	53.5	72.6
		busoga	63.7	55.2	71.3
		kampala	74.5	62.5	83.6
		karamoja	51.1	34.5	67.4
		kigezi	67.6	54.6	78.3
		lango	67.7	57.8	76.3
		north central	59.4	49.5	68.6
		south central	69.8	58.2	79.3
		teso	69.8	62.1	76.5
		tooro	60	52.4	67.2
		west nile	83.9	72.1	91.3
	2011	east	50.5	37.9	63
		east central	25.6	16	38.2
		kampala	59.5	49.3	69
		karamoja	52.4	38.3	66.1
		north	46.5	36.5	56.7
		north central	43.1	32.5	54.3
		south central	40.9	25.2	58.6
		southwest	40.4	30.9	50.7
		west	55.2	45.3	64.7
		west nile	72.1	60.6	81.2
	2006	east	12.3	6.2	22.9
		east central	6.7	3.2	13.3
		kampala	14.7	6.6	29.6
		north	16.4	11.1	23.5
		north central	6.2	1.9	18.3
		south central	4.2	1.4	11.8
		southwest	5.5	1.5	17.8
		west	9.1	4.2	18.6
		west nile	17.2	7.9	33.3

### Country-to-country ITN usage comparison

Because the years in which national DHSs were conducted was different for the four countries of interest, as well as the extensive diversity in national economics, educational systems, urban/rural settings, and regional divisions, comparing ITN usage across countries was not deemed to be useful. Instead, we have compared inequity internationally, among high-burden countries, using recent survey data.

### Inequality by economic status

We have seen inequality between population groups in wealth quintile among pregnant women who slept under ITN the night before the national DHSs. For instance, in Nigeria in 2003, considerable amount of inequality was observed when inequality was measured using both absolute (D and ACI) and relative measures (R and RCI). It was found that ITN utilization was dominant on the richest population group (ACI = 0.1) and (RCI = 11.5). But there was no inequality with summary measures PAR and PAF.

In Nigeria the inequality was also present in the 2008 and 2011 studies. In both years all the applied summary measures (D, PAF, PAR, R, ACI and RCI) imply the existence of inequality among wealth quintiles with the rich population being advantaged (In 2008, D = 2.8, PAF = 5.4, PAR = 0.3, R = 2.2, ACI = 0.6, RCI = 12.8; in 2011, D = 5.5, PAF = 10.2, PAR = 1.7, R = 1.4, ACI = 0.7, RCI = 3.8). In 2013, even though there was no inequality in Nigeria across wealth quintiles according to summary measures PAR and PAF, the other measures indicated presence of slight inequality that advantaged the richest population. For instance, when the value of zero indicates absence of inequality, this result showed D value of 0.9,which means there is inequality that favors towards the rich population. In the case of R, the value of 1 indicates no inequality while our result was 1.1 that showed a slight inequality exists between wealth quintiles.

But during the 2016 and 2018 KDHSs, the opposite scenario has happened where the existed inequality that favored the richest population in 2003, 2008, 2011 and 2013 became reversed and the disadvantaged population ( the poorest quintiles) were advantaged. For instance, in 2016, the absolute summary measures are D = -28.3 and ACI = -5.7 and the relative summary measures are R = 0.4, and RCI = -14.4. The 2018 finding also showed that D was -29.8, R 0.6, ACI and RCI were -6.4 and -11.1, respectively.

In the case of DRC, inequality among wealth quintile groups was present in 2007, 2010, 2013 and 2017. In 2007 and 2010 ITN utilization by pregnant women were dominated by the richest wealth quintile with D = 5.7, PAF = 35.2, PAR = 1.9, R = 4.1, RCI = 20.7 in 2007 and ACI = 1.9, D = 12.6, PAF = 12.2, PAR = 5.2, R = 1.4, RCI = 4.4 in 2010.

The finding from 2013 and 2017 survey also showed the presence of inequality that favored the richest quintile but the extent of the inequality was greater in the year 2017 with PAF and PAR value45.7 and 24.5 respectively.

There was a similar trend also for Uganda where the rich was favored regarding ITN utilization. In 2006 ACI became 1.7, D became 10.2, PAF and PAR were also high, 72.1 and7.3 respectively. In 2011, even though ACI, D and RCI showed that the poorest group was being favored, there was no inequality a cross wealth quintile according to R = 1.0.

But in the year 2016 the existence of inequality was confirmed by both absolute and relative summary measures and we found there was some amount of inequality across wealth quintile that advantaged the richest population group. Some of the summary measures were, ACI = 2.9, D = 16.4, PAF = 18.9, PAR = 12.1, RCI = 4.4.

According to the Mozambique DHSs that was conducted in 2011 and 2015, there was inequality among pregnant women who were sleeping under ITN across wealth quintile groups. For instance in 2011, ACI was 2.1, D was 10.4, PAF was 19.5, PAR was 6.7, and RCI became 6.2. In 2015, the same scenario was also found in 2015 and these summary measure values were higher than that of 2011’s. For instance in 2015, ACI = 3.0, D = 18.0, PAF and PAR were 22.8 and 11.9, respectively. While R was 1.4, RCI was 5.8.

### Inequality by education

In Nigeria, in 2003, according to our summary measure, there was inequality to across educational achievement where those achieved more than secondary school were advantageous with regard to ITN utilization (RCI = 2.4).In 2008 the same scenario has happened that favored those achieved more than secondary school (D = 2.1, PAF = 27.6, PAR = 1.3,R = 1.5, RCI = 10.0). in the year 2011 and 2013, no inequality was observed across educational status with summary measures PAF,PAR and R but minor inequality was observed with D,ACI, and RCI.

In the year 2016 and 2018, the inequality existed in the opposite direction from that of 2003 and 2008. Population groups with no education has been favored with regard to ITN utilization. Some of the summary measures were close to each other in the year 2016 and 2018. For instance, in 2016(D = -20.9, R = 0.6, ACI = -4.9, RCI = -12.4) and in 2018 (D = -24.0, R = 0.6, ACI = -5.8, RCI = -10.0).

In the case of DRC, inequality was present in all study years and it has similar pattern except in 2007 where population with no education were advantaged than those who are above secondary school. In 2007 the ACI, D, RCI were -0.3, -1.5, -6.2, respectively.

But during 2010, 2013 and 2017 those populations who were above secondary school were favored with regard to ITN utilization. For instance, in 2010 PAF and PAR estimates showed that 6.3 and 2.7 respectively. As compared to the 2013 finding, in 2017 greater degree of inequality was found and the population who have achieved above secondary degree was using more ITNs than those with no education. For example, in 2013 PAF was 5.8 and in 2017 it became 17.5. Similarly in 2013 D and PAR were 9.4 and 3.5 while in 2017 they became 27.5 and 9.4 respectively.

Similar finding was found in the case of Uganda and Mozambique where inequality existed and it has favored the educated population. For instance in Uganda in 2006 the summary measures like D = 8.2, PAF = 74.0,PAR = 7.5,R = 1.9 and RCI was found 9.6. similarly in Mozambique, in 2015, ACI = 2.1, D = 12.3, PAF = 15.9,PAR = 8.3 and RCI became 4.0.

### Inequality by place of residency

In Nigeria, in the year 2003 and 2008 there was a inequality that was favoring the rural population with D value being -1.3 and – 0.2 respectively but there was no inequality with R, PAR and PAF measures. In the following study years, 2011 and 2013, the figures were similar and they indicated inequality that favored the urban population with regard to ITN utilization. For instance PAF and PAR resulted were 5.0 and 0.9 in 2011 and 5.1 and 0.8 in 2013.

Again in the year 2016 and 2018 the findings were similar to that of the 2003 and 2008 where the rural population if Nigeria were favored. Even though there was no inequality with summary measures PAF and PAR, D and R showed a significant figure of inequality. For instance in 2016 D = -12.6 and R was 0.7, and in 2018 D was -20.4 with similar R value with that of 2016’s.

In DRC in both 2007 and 2010 inequality that favored the urban population was present but the degree of inequality was minimal in that of 2010 with PAF and PAR value of 1.0 and 0.4 respectively where in 2007 it was 29.6 and 1.6. The result in 2013 was different from other study years and the rural population was favored with regard to ITN utilization among pregnant women based on simple summary measures D (-3.7) and R value (0.9). in the case of the final study year, 2017, the urban population was back to being at the advantaged population group with D = 13.9, PAF = 15.7, PAR = 8.4, R = 1.3.

Similar situation occurred in Uganda and Mozambique throughout the study years even though the degree of inequality varies, pregnant women who belong to the urban population were advantaged than the rural population in both Uganda and Mozambique. For instance in Uganda in in 2011 summary measures had found as follows (D = 9.9, PAF = 18.1, PAR = 8.5,R = 1.2). In the same year in Mozambique D was 16.7, PAF = 35.9, PAR = 12.4, R = 1.6.

### Inequality by subnational regions

Among the subnational regions in Nigeria, those regions that have the highest estimated ITN utilization were favored as compared to those regions with lower estimate. Based on summary measures PAF and PAR, the degree of inequality was higher in the year 2011.

We have seen that the Nigerian DHS and MICS survey used different subnational classification. to see specific regions that were favored, from the DHS figures in 2003 nord-est region was favored with D = 1.7, PAF = 29.2. In 2008, South South region was advantaged summary value measures being D = 3.7,PAF = 48.1, PAR = 2.3,R = 2.1. South east and nord oust were favored during the year 2013 and 2018 respectively. With D = 10.2, PAF = 42.3, PAR = 7.0, R = 1.8 begin for 2013 and D = 49.7, PAF = 36.0, PAR = 20.9, R = 2.7 for 2018.

From the MICS evidence in 2011 Gombe region was advantaged with regard to ITN utilization with summary value measure values as follows,D = 46.8, PAF = 181.8, PAR = 31.3, R = 27.4. In the year 2016, Yobe region were the favored region with D = 62.5, PAF = 68.1, PAR = 27.0,R = 16.5.

In DRC subnational region inequality was also found. Based on the DHS subnational regions classification, we have found that a high degree of inequality across ITN utilization that favored the bas Congo region with D value 24.6, PAF = 350.1, PAR = 19.4 and R value 75.3. And in the year 2013 Bandundu region was advantaged than the other regions of the country. To mention the summary measure values, D = 40.6, PAF = 31.3, PAR = 18.8,R = 2.1.Based on the MICS subnational region classification Maniema and Sud Ubangi regions were favored in the year 2010 and 2017 respectively. The PAF and PAR value were 56.5 and 24.0 in 2010, 56.0 and 30.1 in 2017.

In the case of Uganda, inequality among sub national regions has been found in all study years,2006,2011 and 2016. In 2006 northern region was favored with PAF and PAR values 69.5 and 7.0 respectively. West Nile region was also advantaged region in both 2011 and 2016 with a some amount of lowered level in 2016. The summary measures were D = 46.5, PAF = 53.2, PAR = 25.0 in 2011 and D = 37.8, PAF = 30.4, PAR = 19.6 in 2016.

Nampula and sofala were two regions in Mozambique that were advantaged inthe utilization of ITN among pregnant women during the year 2011 and 2015 respectively. According to the applied summary measures, the degree of inequality decreased in 2015 than in 2011. In 2011 the D, PAF, PAR and R values were 43.5,51.5,17.7 and 6.0 respectively. While in 2015 the D value was 41.0, PAF was 35.4, PAR and R was 18.4 and 2.4.

### Intra-national ITN inequity

We have compared inequity among Nigeria, the DRC, Mozambique, and Uganda– all countries with a high burden of malaria– using the most recent year’s survey. For educational attainment, when we use the five summary measures *D*, PAF, PAR, ACI, and *R*, the DRC had the highest degree of inequity (variability) by all applied measures except ACI, where Nigeria reported the greatest degree of inequity. Uganda exhibited the second highest degree of inequity after the DRC using summary measures PAR and *R*, and Mozambique exhibited the second greatest inequity when using PAF. When inequity was measured using PAF and PAR, Nigeria was free of educational attainment-based inequity (at the most recent study year) but with summary measures ACI, *D* and RCI, it was the country with the leading level of inequity. In the DRC, Mozambique, and Uganda, it was those with no formal education that possessed the lowest ITN usage rates. In Nigeria, those with no formal education had the highest rates of ITN usage.

Regarding urban/rural setting, higher level of inequity (variability) was observed in the DRC followed by Uganda in all summary metrics (PAF, PAR, and *R*) except for D. Mozambique and Nigeria followed, with lower degrees of inequity. For the summary measure *D*, Nigeria exhibited the highest inequity, followed by the DRC, Uganda, and Mozambique. In all countries except Nigeria, pregnant women who resided in urban areas were more likely to use ITNs.

Inequality across wealth quintile was also compared within the four countries. When inequality was measured by summary metrics, ACI, *D*, PAF, PAR, *R*, and RCI, the DRC exhibited the highest degree of internal inequality (variability) in ITNs use by economic status. Using summary metrics ACI, *D*, and RCI, Nigeria, Mozambique and Uganda exhibited the second, third and fourth most variability, respectively. In all countries except Nigeria, ITN usage favored the wealthiest quintile of society; however, in Nigeria, it was the poorest quintile that had the highest rate of ITN usage.

When we compare inequity(variability) by subnational regions, the DRC has the greatest internal inequity according to summary metrics (D,PAF,PAR and R). The DRC was followed by Nigeria, Mozambique, and Uganda, in that order.

Table 6 Most recent year ITN utilization among pregnant women, by dimension and summary metrics.

**Table 6:** Most recent year ITN utilization among pregnant women, by dimension and summary metric

Country	Year	Dimension	Summary Metric	Proportion of ITN Usage (%)	95% CI (Lower Bound)	95% CI (Upper Bound)
Nigeria	2018	Economic status	ACI	-6.4	-7.5	-5.4
Nigeria	2018	Economic status	D	-29.8	-35.9	-23.6
Nigeria	2018	Economic status	PAF	0.0	-4.4	4.4
Nigeria	2018	Economic status	PAR	0.0	-2.6	2.6
Nigeria	2018	Economic status	R	0.6	0.5	0.6
Nigeria	2018	Economic status	RCI	-11.1	-11.5	-10.7
Nigeria	2018	Educational attainment	ACI	-5.8	-6.8	-4.8
Nigeria	2018	Educational attainment	D	-24.0	-28.1	-19.8
Nigeria	2018	Educational attainment	PAF	0.0	-2.9	2.9
Nigeria	2018	Educational attainment	PAR	0.0	-1.7	1.7
Nigeria	2018	Educational attainment	R	0.6	0.6	0.7
Nigeria	2018	Educational attainment	RCI	-10.0	-10.3	-9.6
Nigeria	2018	Rural/Urban Setting	D	-20.4	-24.6	-16.2
Nigeria	2018	Rural/Urban Setting	PAF	0.0	-2.0	2.0
Nigeria	2018	Rural/Urban Setting	PAR	0.0	-1.2	1.2
Nigeria	2018	Rural/Urban Setting	R	0.7	0.6	0.8
Nigeria	2018	Subnational region	D	49.7	42.7	56.7
Nigeria	2018	Subnational region	PAF	36.0	27.6	44.4
Nigeria	2018	Subnational region	PAR	20.9	16.0	25.8
Nigeria	2018	Subnational region	R	2.7	2.2	3.4
DRC	2017	Economic status	ACI	6.5	4.8	8.3
DRC	2017	Economic status	D	42.2	32.3	52.1
DRC	2017	Economic status	PAF	45.7	39.1	52.3
DRC	2017	Economic status	PAR	24.5	21.0	28.1
DRC	2017	Economic status	R	2.2	1.8	2.7
DRC	2017	Economic status	RCI	12.2	11.5	12.9
DRC	2017	Educational attainmental attainment	ACI	5.5	3.9	7.1
DRC	2017	Educational attainment	D	27.5	19.7	35.4
DRC	2017	Educational attainment	PAF	17.5	9.6	25.3
DRC	2017	Educational attainment	PAR	9.4	5.2	13.6
DRC	2017	Educational attainment	R	1.8	1.5	2.2
DRC	2017	Educational attainment	RCI	10.3	9.7	10.9
DRC	2017	Rural/Urban Setting	D	13.9	6.1	21.6
DRC	2017	Rural/Urban Setting	PAF	15.7	12.7	18.7
DRC	2017	Rural/Urban Setting	PAR	8.4	6.8	10.0
DRC	2017	Rural/Urban Setting	R	1.3	1.1	1.5
DRC	2017	Subnational region	D	62.7	48.7	76.6
DRC	2017	Subnational region	PAF	56.0	40.3	71.8
DRC	2017	Subnational region	PAR	30.1	21.6	38.5
DRC	2017	Subnational region	R	4.0	2.5	6.4
Mozambique	2015	Economic status	ACI	3.0	0.3	5.8
Mozambique	2015	Economic status	D	18.0	3.2	32.8
Mozambique	2015	Economic status	PAF	22.8	9.5	36.1
Mozambique	2015	Economic status	PAR	11.9	4.9	18.8
Mozambique	2015	Economic status	R	1.4	1.0	1.9
Mozambique	2015	Economic status	RCI	5.8	5.2	6.3
Mozambique	2015	Educational attainment	ACI	2.1	-0.3	4.5
Mozambique	2015	Educational attainment	D	12.3	-0.6	25.2
Mozambique	2015	Educational attainment	PAF	15.9	3.6	28.2
Mozambique	2015	Educational attainment	PAR	8.3	1.9	14.7
Mozambique	2015	Educational attainment	R	1.3	1.0	1.6
Mozambique	2015	Educational attainment	RCI	4.0	3.6	4.4
Mozambique	2015	Rural/Urban Setting	D	6.3	-4.4	17.0
Mozambique	2015	Rural/Urban Setting	PAF	8.3	3.3	13.3
Mozambique	2015	Rural/Urban Setting	PAR	4.3	1.7	6.9
Mozambique	2015	Rural/Urban Setting	R	1.1	0.9	1.4
Mozambique	2015	Subnational region	D	41.0	25.1	56.9
Mozambique	2015	Subnational region	PAF	35.4	11.1	59.6
Mozambique	2015	Subnational region	PAR	18.4	5.8	31.1
Mozambique	2015	Subnational region	R	2.4	1.5	3.7
Uganda	2016	Economic status	ACI	2.9	1.4	4.3
Uganda	2016	Economic status	D	16.4	8.5	24.3
Uganda	2016	Economic status	PAF	18.9	12.3	25.4
Uganda	2016	Economic status	PAR	12.1	7.9	16.4
Uganda	2016	Economic status	R	1.3	1.1	1.4
Uganda	2016	Economic status	RCI	4.4	4.3	4.6
Uganda	2016	Educational attainment	ACI	3.0	1.6	4.3
Uganda	2016	Educational attainment	D	19.1	8.3	29.9
Uganda	2016	Educational attainment	PAF	13.5	1.7	25.3
Uganda	2016	Educational attainment	PAR	8.7	1.1	16.3
Uganda	2016	Educational attainment	R	1.4	1.1	1.6
Uganda	2016	Educational attainment	RCI	4.6	4.4	4.8
Uganda	2016	Rural/Urban Setting	D	8.5	1.7	15.3
Uganda	2016	Rural/Urban Setting	PAF	10.1	8.3	11.9
Uganda	2016	Rural/Urban Setting	PAR	6.5	5.3	7.7
Uganda	2016	Rural/Urban Setting	R	1.1	1.0	1.3
Uganda	2016	Subnational region	D	37.8	24.4	51.2
Uganda	2016	Subnational region	PAF	30.4	18.9	41.9
Uganda	2016	Subnational region	PAR	19.6	12.1	27.0
Uganda	2016	Subnational region	R	1.8	1.4	2.3

## Discussion

The use of ITNs is one of the most effective and efficient strategies used to prevent malaria infection during pregnancy [25]. ITNs have also been shown to reduce malaria episodes among children underfive years of age by approximately 50%, and to reduce all-cause mortality by 17% globally [26]. This study examined economic, educational, urban/rural setting, and subnational geographic determinants of ITN usage among pregnant women in the high-malaria-burden countries of Nigeria, the DRC, Mozambique, and Uganda. The findings from our study show that being in the highest wealth quantile, attaining higher educational level and living in urban areas increased the likelihood of ITN usage.

Based on Difference as a measure of absolute health inequality, the economic status-based inequality assessment indicated that ITNis more utilized in economically better-off women in most DHS time points for Nigeria, DRC, Uganda and Mozambique. Similar findings were documented in Kenya and Tanzania [27, 28]. This shows disproportionate malaria risk meaning the risk of being bitten by a mosquito is way higher in the poor. Although, slight inequality was observed by complex measures (PAR, PAF) in all five surveys in Nigeria, in terms of PAR, ITNutilization in the advantaged survey populations across all countries is still higher than the national average. In case of PAF, the difference between advantaged groups in terms of ITNutilization is five times the national average in Nigeria in 2008 but became ten times in 2011. The same is true for DRC, where PAF measures increased from being thirty-five to forty-five times the national average in 2007 and 2017 respectively. This shows that there's a disproportionate risk of malaria among both the rich and poor. Inequalities like these should be taken into consideration while implementing interventions in view of risk based deployment of interventions like health education targeting low economic and education groups.

Our analysis has shown that ITN utilization was generally highest among the wealthiest quintiles, but usage increased across survey years for most economic groups. This finding corroborates studies from Kenya, Tanzania, and Uganda, which found that poverty was an impediment to the purchase of mosquito nets both untreated and treated [9, 27, 29]. The poorer economic strata were less likely to own an untreated net, acquire an ITN, or express a desire to pay for one. Charging for nets has been linked to high levels of adoption (up to 50%) among the lowest economic quintile, but this has only happened in regions where nets have been aggressively marketed [30]. Despite this finding, data analyzed in this study from Nigeria and Uganda indicated that economically disadvantaged pregnant women were more likely to utilize ITNs. ITN usage disproportionately benefit the poor since the wealthy are more likely to live in less malaria-prone areas,with less risks as a result of completed houses, covered eaves that minimize mosquito exposure, glass windows and/or screens. Poorer households are more likely to live in areas with inadequate drainage systems where mosquito breeding sites are abundant, and in close proximity to livestock, making household members more vulnerable to mosquito bites and raising the perceived need for ITN usage [31, 32]. In a recent study from Mozambique, families in the poorest wealth tertile (33.3%) had higher likelihoods of sleeping under an ITN (AOR 2.36; 95^th^ percentile CI: 1.16–4.81) compared to the wealthiest ones [33]. Because of their economic advantage, the wealthy may be more likely to investigate and employ malaria preventive measures such as indoor residual spraying (IRS) or the use of insect repellents [25, 32]. In contrast, these alternatives may be prohibitively costly for poorer pregnant women, resulting in a preference for a freely-provided ITN [33].

In all the years except for 2007, inequalities were observed across Nigeria, the DRC, Mozambique, and Uganda by educational attainment (± secondary education).. Recent studies from Kenya and Ethiopia lend credence to this finding, where the highest proportion of ITN users were women who were more highly educated. Enrollment in higher education or attaining secondary level of education was found to determine ITN usage by positively influencing care seeking behavior and use of ITN in pregnancy [34]. Some African studies have also shown a link between education and increased usage of ITNs and other vector control strategies [35, 36].

In contrast, a cross-sectional survey from Tanzania reported no link between education level and use of ITNs [37]. In Nigeria, a recent study found no significant relationship between degree of education (*p* = 0.269) and use of ITNs [38]. On the other hand, a study from Ethiopia found that increasing educational attainment was a statistically significant positive explanatory factor for the use of ITNs among pregnant women. Extrapolating these findings to Ethiopia's rural areas, where approximately 85% of the population has relatively lower educational attainment, ITN utilization is expected to be much lower in these sections of the nation [39]. This finding suggests that a greater degree of education may be necessary to influence malaria preventive and control intervention uptake [36, 40]. It is important to note that more qualitative research is needed regarding the reasons for ITN use. Without such data, it is difficult to determine if ITN usage is simply a by-product of supply chain logistics, i.e. where they are being sent, and proximity. It is equally difficult to disentangle the causes of ITN usage when factors that are associated with ITN usage are often interrelated where wealthy people probably have higher education levels and may be more likely to live in urban settings. urban–rural setting has been linked with ITN usage, as has educational attainment;, however, the actual causative agent, urban/rural setting, educational attainment, a combination of the two, or an unknown third component, is more difficult to assess.

In our analysis of survey data from the DRC, ITN usage was positively associated with educational attainment in all survey years except for 2007, when educational attainment was negatively correlated with ITN usage. In a study conducted in the DRC at approximately the same time (2007), it was found that women with a high school degree or higher were 1.3 times more likely to use ITNs (OR = 1.3; 95^th^ percentile CI: 1.085–1.611) compared to those with only a primary school education [41]. Increased education, particularly for young women, has been linked to a number of positive health outcomes, including lower newborn and maternal mortality, in this study as well as others from Sub-Saharan Africa [41].

Our results suggest that in Nigeria in the years 2003, 2008, 2016, and 2018, rural pregnant women were more likely than urban pregnant women to use ITNs.This supports previous findings that investigated variation in ITN utilization between rural and urban pregnant women in Nigeria. In that study, 86.1% of rural residents utilized ITNs, compared with 74.1% among urbanites [42]. Similarly, in Cameroon and Sierra Leone, it was discovered that, following a countrywide mass distribution of ITNs, rural residents were more likely to adopt regular ITN usage compared to urban dwellers [43]. Finally, in a study similar to ours, that extracted data from the Ghana 2014 DHS and the 2016 Malaria Indicator Survey, it was concluded that, in both survey years, increasing net usage was associated with living in rural settings [31]. The possible explanation for this might be, in most countries, the malaria burden is higher in rural areas compared to urban settings and therefore intervention coverage is higher in rural settings.

Conversely, many recent studies from Nigeria [44] and other sub-Saharan African countries, such as Ghana [45], Equatorial Guinea [46] and Senegal [26] have reported greater ITN usage in urban areas. A Malawian cross-sectional research of 528 respondents also indicated that after a social marketing campaign, households generally owned more ITNs, but in urban areas, the rate grew to 29%, compared to 6.4% in rural areas [47]. Women in rural areas were similarly less likely to use ITNs.This could be due to an imbalance in ITN outlets favoring urban areas, a lack of education in rural areas, difficulty in creating marketing campaigns in rural settings, or a lack of the economic resources to purchase ITNs in rural regions [40]. In areas where rural residents have possessed ITNs in Nigeria and Tanzania, studies in the past years have reported that actual utilization remains low [48, 49]. It is imperative that in addition to the collection of more robust qualitative data from survey participants, and more complex statistical models that tease out causality, that geographic mapping of distribution sites is incorporated, so that researchers can assess the influence of simple accessibility. Once there is a better understanding of causes of ITN usage, and differences between countries and environments, subnational tailoring of resource allocation can be much more effective.

## Conclusion

The observed difference in ITN use across all dimensions of inequality in all the four countries was highly pronounced. ITN utilization was generally highest among the wealthiest quintiles. There was an association between ITN usage and increasing educational attainment. Similar finding was reported for subnational regions. There was an overall increasing trend of ITN use over the survey times in all the four countries. This calls for targeted intervention by stakeholders to improve ITN use among pregnant women. Tailored combination of cost-effective interventions could be considered to achieve synergy and maximize the gains.

## Data Availability

The datasets generated and/or analysed during the current study are available in the WHO HEAT version 3.1 softwarerepository (https://whoequity.shinyapps.io/HEAT/).
